# Clinical, cognitive and neuroanatomical associations of serum NMDAR autoantibodies in people at clinical high risk for psychosis

**DOI:** 10.1038/s41380-020-00899-w

**Published:** 2020-10-19

**Authors:** Thomas A. Pollak, Matthew J. Kempton, Conrad Iyegbe, Angela Vincent, Sarosh R. Irani, Ester Coutinho, David A. Menassa, Leslie Jacobson, Lieuwe de Haan, Stephan Ruhrmann, Gabriele Sachs, Anita Riecher-Rössler, Marie-Odile Krebs, Paul Amminger, Birte Glenthøj, Neus Barrantes-Vidal, Jim van Os, Bart P. F. Rutten, Rodrigo A. Bressan, Mark van der Gaag, Robert Yolken, Matthew Hotopf, Lucia Valmaggia, James Stone, Anthony S. David, The EUGEI High-Risk Study, Maria Calem, Stefania Tognin, Gemma Modinos, Lieuwe de Haan, Mark van der Gaag, Eva Velthorst, Tamar C. Kraan, Daniella S. van Dam, Nadine Burger, Barnaby Nelson, Patrick McGorry, Christos Pantelis, Athena Politis, Joanne Goodall, Stefan Borgwardt, Sarah Ittig, Erich Studerus, Renata Smieskova, Ary Gadelha, Elisa Brietzke, Graccielle Asevedo, Elson Asevedo, Andre Zugman, Araceli Rosa, Anna Racioppi, Manel Monsonet, Lídia Hinojosa-Marqués, Thomas R. Kwapil, Mathilde Kazes, Claire Daban, Julie Bourgin, Olivier Gay, Célia Mam-Lam-Fook, Dorte Nordholm, Lasse Randers, Kristine Krakauer, Louise Glenthøj, Merete Nordentoft, Dominika Gebhard, Julia Arnhold, Joachim Klosterkötter, Iris Lasser, Bernadette Winklbaur, Philippe A. Delespaul, Jim van Os, Philip McGuire

**Affiliations:** 1 Department of Psychosis Studies, Institute of Psychiatry, Psychology and Neuroscience, King’s Health Partners, King’s College London, London, UK; 2 Precision Psychiatry Cluster, National Institute for Health Research Biomedical Research Centre at South London and Maudsley NHS Foundation Trust and King’s College London, London, UK; 3 South London and Maudsley NHS Foundation Trust, London, UK; 4 Nuffield Department of Clinical Neurosciences, John Radcliffe Hospital, Oxford, UK; 5 Autoimmune Neurology Group, Nuffield Department of Clinical Neurosciences, John Radcliffe Hospital, Oxford, UK; 6 Division of Neuroscience, Institute of Psychiatry, Psychology and Neuroscience, King’s Health Partners, King’s College London, London, UK; 7 Biological Sciences, Faculty of Environmental and Life Sciences, University of Southampton, Southampton, UK; 8 Amsterdam UMC, University of Amsterdam, Psychiatry, Department Early Psychosis, Meibergdreef 9, Amsterdam, The Netherlands; 9 Department of Psychiatry and Psychotherapy, Faculty of Medicine and University Hospital, University of Cologne, Cologne, Germany; 10 Medical University of Vienna, Department of Psychiatry and Psychotherapy, Vienna, Austria; 11 Medical Faculty, University of Basel, Basel, Switzerland; 12 University of Paris, GHU-Paris, Sainte-Anne, C’JAAD, Hospitalo-Universitaire department SHU, Inserm U1266, Institut de Psychiatrie (CNRS 3557), Paris, France; 13 Centre for Youth Mental Health, University of Melbourne, 35 Poplar Road (Locked Bag 10), Parkville, VIC 4853052 Australia; 14 Orygen, The National Centre of Excellence in Youth Mental Health, The University of Melbourne, Parkville, VIC Australia; 15 Centre for Neuropsychiatric Schizophrenia Research (CNSR) & Centre for Clinical Intervention and Neuropsychiatric Schizophrenia Research (CINS), Mental Health Centre Glostrup, University of Copenhagen, Glostrup, Denmark; 16 Departament de Psicologia Clínica I de la Salut (Universitat Autònoma de Barcelona), Fundació Sanitària Sant Pere Claver (Spain), Spanish Mental Health Research Network (CIBERSAM), Barcelona, Spain; 17 Department of Psychiatry, UMC Utrecht Brain Centre, Utrecht, The Netherlands; 18 Department of Psychiatry and Neuropsychology, School for Mental Health and Neuroscience, Mental Health Research and Teaching Network, Maastricht University Medical Centre, P.O. Box 616, 6200 MD 464, Maastricht, The Netherlands; 19 PRISMA Clinic, Y-MIND—Institute for Prevention of Mental Disorders (PRISMA); LiNC—Lab Interdisciplinar Neurociências Clínicas, Escola Paulista de Medicina, Universidade Federal de São Paulo—UNIFESP, Sao Paulo, Brazil; 20 VU University, Faculty of Behavioural and Movement Sciences, Department of Clinical Psychology and Amsterdam Public Mental Health research institute, van der Boechorststraat 7, 1081 BT Amsterdam, The Netherlands; 21 Stanley Neurovirology Division. Department of Pediatrics, Johns Hopkins School of Medicine, Baltimore, MD USA; 22 Department of Psychological Medicine, Institute of Psychiatry, Psychology and Neuroscience, King’s College London, London, UK; 23 Department of Psychology, Institute of Psychiatry, Psychology & Neuroscience, King’s College London, London, UK; 24 Department of Neuroimaging, Centre for Neuroimaging Sciences, Institute of Psychiatry, Psychology and Neuroscience, King’s College London, London, UK; 25 Institute of Mental Health, University College London, Maple House, 149 Tottenham Court Road, London, W1T 7NF UK; 26 AMC, Academic Psychiatric Centre, Department Early Psychosis, Meibergdreef 5, 1105 AZ Amsterdam, The Netherlands; 27 Icahn School of Medicine at Mount Sinai, Department of Psychiatry, 1425 Madison Avenue, New York, NY 10029 USA; 28 Parnassia Psychiatric Institute, Department of Psychosis Research, Zoutkeetsingel 40, 2512 HN The Hague, The Netherlands; 29 Department of Psychiatry, University of Basel, Basel, Switzerland; 30 Department of Psychiatry and Psychotherapy, University of Lübeck, Lübeck, Germany; 31 University Psychiatric Hospital, Wilhelm Klein-Strasse 27, 4002 Basel, Switzerland; 32 University of Basel, Department of Psychology, Division of Personality and Developmental Psychology, Basel, Switzerland; 33 Depto Psiquiatria, Escola Paulista de Medicina, Universidade Federal de São Paulo—UNIFESP, Sao Paulo, Brazil; 34 Departament de Biologia Evolutiva, Ecologia i Ciències Ambientals (Universitat de Barcelona), Barcelona, Spain; 35 Departament de Psicologia Clínica i de la Salut (Universitat Autònoma de Barcelona), Barcelona, Spain; 36 Department of Psychology, University of Illinois at Urbana-Champaign, Champaign, IL USA; 37 University Paris Descartes, Hôpital Sainte-Anne, C’JAAD, Service Hospitalo-Universitaire, Inserm U894, Institut de Psychiatrie (CNRS 3557), Paris, France; 38 Mental Health Center Copenhagen and Center for Clinical Intervention and Neuropsychiatric Schizophrenia Research, CINS, Mental Health Center Glostrup, Mental Health Services in the Capital Region of Copenhagen, University of Copenhagen, Copenhagen, Denmark; 39 Psyberlin, Berlin, Germany; 40 Mondriaan Mental Health Trust, Heerlen, The Netherlands

**Keywords:** Schizophrenia, Neuroscience, Prognostic markers

## Abstract

Serum neuronal autoantibodies, such as those to the NMDA receptor (NMDAR), are detectable in a subgroup of patients with psychotic disorders. It is not known if they are present before the onset of psychosis or whether they are associated with particular clinical features or outcomes. In a case–control study, sera from 254 subjects at clinical high risk (CHR) for psychosis and 116 healthy volunteers were tested for antibodies against multiple neuronal antigens implicated in CNS autoimmune disorders, using fixed and live cell-based assays (CBAs). Within the CHR group, the relationship between NMDAR antibodies and symptoms, cognitive function and clinical outcomes over 24 month follow-up was examined. CHR subjects were not more frequently seropositive for neuronal autoantibodies than controls (8.3% vs. 5.2%; OR = 1.50; 95% CI: 0.58–3.90). The NMDAR was the most common target antigen and NMDAR IgGs were more sensitively detected with live versus fixed CBAs (*p* < 0.001). Preliminary phenotypic analyses revealed that within the CHR sample, the NMDAR antibody seropositive subjects had higher levels of current depression, performed worse on the Rey Auditory Verbal Learning Task (*p* < 0.05), and had a markedly lower IQ (*p* < 0.01). NMDAR IgGs were not more frequent in subjects who later became psychotic than those who did not. NMDAR antibody serostatus and titre was associated with poorer levels of functioning at follow-up (*p* < 0.05) and the presence of a neuronal autoantibody was associated with larger amygdala volumes (*p* < 0.05). Altogether, these findings demonstrate that NMDAR autoantibodies are detectable in a subgroup of CHR subjects at equal rates to controls. In the CHR group, they are associated with affective psychopathology, impairments in verbal memory, and overall cognitive function: these findings are qualitatively and individually similar to core features of autoimmune encephalitis and/or animal models of NMDAR antibody-mediated CNS disease. Overall the current work supports further evaluation of NMDAR autoantibodies as a possible prognostic biomarker and aetiological factor in a subset of people already meeting CHR criteria.

## Introduction

Neuronal surface autoantibodies (NSAbs) have been identified in blood samples from a minority of patients with psychotic disorders and several other unrelated neurological and psychiatric conditions [1–3]. These autoantibodies target cell-surface proteins, such as the NMDA receptor (NMDAR), gamma-aminobutyric acid_A_ (GABA_A_) or GABA_B_ receptor, α-amino-3-hydroxy-5-methyl-4-isoxazolepropionic acid (AMPA) receptor, leucine-rich glioma-inactivated 1 (LGI1) or contactin-associated protein-like 2 (CASPR2) or intracellular proteins, such as glutamic acid decarboxylase (GAD). When present in cerebrospinal fluid (CSF), these IgG-subclass cell surface-targeting antibodies are considered pathogenic and associate with severe autoimmune encephalopathy syndromes, which can present with varied psychiatric features, particularly in the case of NMDAR antibody encephalitis, plus impaired cognitive function and pathological changes centred around limbic brain regions [4–8]. Estimates of the prevalence of NSAbs in patients with psychosis vary depending on the age, condition studied and the assay used. Live cell-based assays (CBAs) consistently detect higher rates than fixed CBAs, and fixed CBA positivity rates vary widely [1, 9–11]. Therefore, the clinical relevance of these serum-only antibodies remains unclear.

At present, there are no data on whether NSAbs are present prior to the onset of psychosis and, if so, whether they predict clinical outcomes. People at clinical high risk (CHR) for psychosis experience subthreshold psychotic symptoms, display memory impairments and show alterations in the structure of limbic brain areas [12–14], findings that show a partial resemblance to those seen in NMDAR antibody-mediated encephalitis [4, 15–17].

Outside of the autoimmune encephalitis context there is increasing evidence that NMDAR antibodies may be associated with cognitive impairment (e.g., in cancer [18, 19], or following viral encephalitis [20]) but this association has not been examined in the context of psychosis-spectrum disorders. In patients who have developed psychosis, most studies have not found clinically significant differences in symptom profile between seropositive and seronegative patients [3, 9, 10, 21], raising potential doubts over their pathogenic relevance. However, in vitro, serum NMDAR antibodies from patients with psychosis disrupt receptor dynamics [22, 23] and function [9, 24], suggesting that the antibodies have pathogenic potential at a molecular level.

It is known that NR1-specific intrathecal B cells—which have not been reported in psychiatric disorders—are a hallmark of NMDAR antibody encephalitis [25] and that circulating B cells can produce these antibodies [26]. The clinical significance of serum autoantibodies may depend on the presence of additional factors, such as blood–brain barrier (BBB) disruption [9, 27], which could allow them access to the CNS. There is widespread evidence for BBB disruption in psychotic disorders [28], although human studies are limited by the requirement for CSF samples or the use of proxy clinical markers such as a history of brain injury. One such marker is the astrocytic calcium-binding protein S100B, which is elevated in schizophrenia [28–31], and can be measured in serum samples alongside autoantibodies.

If autoantibodies to the NMDAR play an aetiological role in some cases of psychosis, one would expect that they would be present before the full clinical expression of the disorder. In all, 10–35% of patients with CHR may subsequently develop psychosis, with the proportion varying between samples [32, 33], and there is an unmet clinical need for biomarkers that can predict which individuals will later become psychotic [34]. Therefore, we sought to address this by investigating various NSAbs in people who are at CHR for psychosis and examining the relationship between NMDAR antibodies and clinical outcomes, cognitive function and psychopathology in CHR subjects.

## Materials and methods

### Samples

Sera were taken from 254 subjects from the EU-GEI high-risk study who met the PACE criteria for the CHR state [35], and from 116 healthy controls (HC). CHR subjects were recruited from 11 centres (London, Amsterdam, The Hague, Copenhagen, Vienna, Basel, Cologne, Melbourne, Paris, Barcelona, and São Paulo) and were reassessed at a timepoint as close to 12 and 24 months as possible. Exclusion criteria were presence of a current or past psychotic disorder, symptoms relevant for inclusion being explained by a medical disorder or drugs or alcohol dependency or IQ < 60.

One-hundred sixteen HC subjects, frequency matched by age and sex, were recruited from the EU-GEI high-risk study (*n* = 55), and the South East London Community Health (SELCOH) study [36] (*n* = 61). HCs from the EU-GEI study were excluded if they met criteria for the CHR state, while HCs from SELCOH were excluded if they screened positive on the Psychosis Screening Questionnaire [37]. Ethical approval for the study was obtained from the local research ethics committees at each of the sites. All participants provided written informed consent.

All serum samples were stored at −80 degrees Celsius until assayed and tested blind to case–control status.

### Phenotype data

All subjects completed a sociodemographic schedule. Baseline psychopathology was assessed using the Comprehensive Assessment of At-Risk Mental States (CAARMS) [38], Brief Psychiatric Rating Scale (BPRS) [39], Scale for the Assessment of Negative Symptoms (SANS) [40], Young Mania Rating Scale, (YMRS) [41], and the Montgomery-Åsberg Depression Rating Scale (MADRS) [42]. The Rey Auditory Verbal Learning Task [43] was used to assess verbal memory function and IQ was estimated using the shortened Wechsler Adult Intelligence Scale III [44]. Overall social functioning was assessed using the Global Assessment of Functioning (GAF) scale. Participants were invited for face-to-face follow-up meetings at 1 year and 2 years after baseline. Where face-to-face meetings were not possible, participants were followed up for 2 years using available clinical records, and this follow-up was extended when additional clinical data was available.

### Immunoassays

Sera were tested for antibodies of the IgG, IgA and IgM subtypes to 32 different substrates (see Supplementary Appendix 1 for a full list), by indirect immunofluorescence on cell-based assays using fixed recombinant HEK293 cells and on frozen sections of rodent and monkey brain tissue (hippocampus and cerebellum) at Euroimmun AG, Lübeck, Germany, as described previously [45]).

Live CBA autoantibody testing for the detection of IgG antibodies to NMDAR was performed using HEK293 cells (European Collection of Authenticated Cell Cultures (ECACC), Public Health England) at the Nuffield Department of Clinical Neurosciences, University of Oxford as described previously [46] with some modifications. Specifically, intensity of fluorescence of each sample was rated on a semi-quantitative visual scoring system of 0–4. Scores of 0 and 1 were rated as negative, 1.5 as low positive and 2–4 as positive. All positive samples were confirmed at dilutions of 1:20, 1:100 and 1:500 using an IgG-Fc specific secondary antibody (goat antihuman Fc IgG; Thermo Scientific 31125), then tested for non-specific binding using HEK293 cells that had been transfected with another antigen (dopamine D2 receptor). The titre of the antibody was given as the dilution of serum giving a score of 1. IgG-positive samples were tested in the Oxford laboratory for binding to live, cultured rat hippocampal neurons as described previously [47]. All cell lines are regularly tested for *Mycoplasma* contamination using immunofluorescence.

Levels of S100B, a putative marker of blood–brain barrier disruption, were assessed using a chemiluminescence assay (Diasorin). High-sensitivity C-reactive protein (hsCRP) was measured as a general marker of systemic inflammation, using high-sensitivity enzyme immunoassays as previously described [48].

### Neuroimaging analysis

MR Images were acquired using the ADNI-2 T1 weighted volumetric sequence (http://adni.loni.usc.edu/methods/documents/mri-protocols/) from 159 CHR subjects on 3T scanners. The ADNI-2 sequence has been specifically developed for multicentre studies and has been optimised to give similar contrast across MRI scanners. Nevertheless, the scanner was modelled as a factor in the statistical analysis to control for this. T1 weighted volumes were and preprocessed using Freesurfer version 6.0.0, using a standard pipeline for quantitation of subcortical volumes (described in full on the FreeSurferWiki page (https://surfer.nmr.mgh.harvard.edu/fswiki). First, intensity correction and skull stripping were performed, followed by segmentation of grey and white matter along with segmentation of subcortical structures in which subject scans are warped to a training set atlas and individual voxels labelled using a Gaussian classifier analysis, yielding maps of deep grey matter, white matter, and CSF structures.

Analyses were performed on Freesurfer’s subcortical segmentation output volumes and estimates of total intracranial volumes. Analysis was a priori restricted to the hippocampus and the amygdala (both bilaterally), as these are the areas that are predominantly affected in autoimmune encephalopathies associated with NSAbs, and these areas have been independently linked to the later onset of psychosis in neuroimaging studies of clinical high-risk populations [49, 50].

### Statistical analysis

Statistical analyses were performed using SPSS 23. Demographic variables were compared between cases and controls and between seropositive and seronegative CHR subjects with independent samples *t*-tests and Mann–Whitney *U-*tests for continuous variables, and chi square and Fisher’s exact tests for categorical data. Based on previous work on neuronal autoantibody prevalence in first episode psychosis populations [10], sample sizes were selected to have statistical power to detect differences.

For symptom and cognitive scales analyses of variance was performed within the General Linear Model with NMDAR antibody status (positive or negative), gender and ethnicity as factors and with age as covariate, after homogeneity of variance was confirmed. Given the polymorphic psychiatric presentation in NMDAR antibody encephalitis [7, 51] we aimed to look at associations with a broad range of psychopathology rather than restricting analyses to any one symptom domain or dimension. In addition to total scores for the CAARMS (whole-instrument score), YMRS, MADRS and SANS, a positive symptoms factor was computed based on the BPRS components analysis of Dingemans et al. [52].

For dichotomous outcome measures (transition, nonremission from CHR state), logistic regression was performed with NMDAR antibody status (positive or negative), age, gender and ethnicity as covariates.

To compare subcortical ‘limbic’ volumes between seropositive and seronegative CHR subjects multivariate analysis of variance with NSAb status (positive or negative), MRI site, gender and ethnicity as factors, and total intracranial volume (TICV) and age as covariates, was performed in the General Linear Model. All main effects were included in our models and the analysis of interactions was a priori limited to the interaction between NSAb status and S100B status.

Significance threshold was set to *p* < 0.05; mean ± sd presented. Owing to the exploratory nature of the analyses, significance values are given for two-tailed tests where applicable and uncorrected for multiple comparisons.

## Results

### Participants

Demographic and clinical information is presented in Table 1. Cases and controls differed in ethnicity, with fewer black participants in the CHR group (8.7%) than in the healthy control group (20.7%). The groups did not differ on any other demographic variable. There was no difference in mean serum S100B level between CHR subjects and controls (*p* = 0.833); CHR subjects had slightly higher baseline inflammation as indexed by hsCRP (*p* = 0.048). Smoking and antipsychotic usage were more frequent in cases than in controls.

**Table 1:** Demographic/basic clinical information and NSAb serostatus by group.

	Total CHR cohort (*n* = 254)	Total HC cohort (*n* = 116)	*p*
Age	22.70 ± 5.00 (range 14 to 45)	23.54 ± 3.38 (range 17 to 34)	0.057
Sex [M: *n* (%)]	136 (53.5)	60 (51.7)	0.822
Ethnicity [*n* (%)]	White 178 (70.4), black 22 (8.7), other 53 (20.9)	White 76 (65.5), black 24 (20.7), other 16 (13.8)	**0.003**
Smoker^a^ [*n* (%)]	133 (53.4)	31 (26.7)	**<0.001**
BMI	24.31 ± 5.30	23.70 ± 4.44	0.288
Current antipsychotic use [*n* (%)]	24 (9.4)	0 (0)	**<0.001**
S100B (ug/L)^b^	0.040 ± 0.049	0.050 ± 0.071	0.833
CRP (mg/L)^b^	1.23 ± 1.08	0.95 ± 0.95	**0.048**
Any antibody (fixed CBA) [*n* (%)]	21 (8.3)	6 (5.2)	0.390
IgG [*n* (%)]	9 (3.5)	4 (3.4)	1.000
IgA [*n* (%)]	8 (3.1)	1 (0.9)	0.283
IgM [*n* (%)]	5 (2.0)	1 (0.9)	0.670
NMDAR antibody (fixed CBA) [*n* (%)]	11 (4.3)	2 (1.7)	0.360
NMDAR IgG [*n* (%)]	1 (0.4)	1 (0.9)	0.529
NMDAR IgA [*n* (%)]	6 (2.4)	0 (0.0)	0.183
NMDAR IgM [*n* (%)]	4 (1.6)	1 (0.9)	1.000
NMDAR IgG (live CBA) [*n* (%)]	13 (5.1)	6 (5.2)	1.000

Bold values indicate statistical significance *p* < 0.05.

*CHR* clinical high risk, *HC* healthy control, *CBA* cell-based assay, *S100B* S100 calcium-binding protein B, *CRP* C-reactive protein, *NMDAR* N-methyl-d-aspartate receptor.

^a^Data available for 365 of 370 subjects.

^b^Adjusted for BMI, gender, age, ethnicity and smoking status.

### Prevalence of NSAbs detected using commercial, fixed CBA and live CBA

**Fig. 1:**
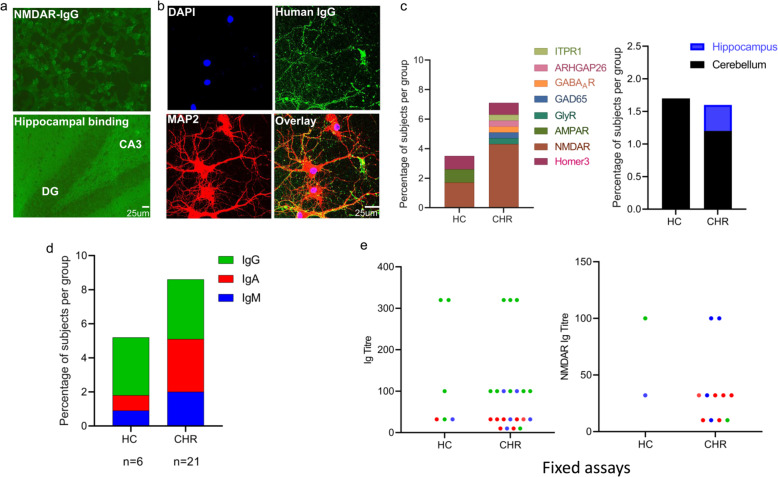
NSAb seroprevalence and associations of seropositivity in CHR and HCs—antibodies detected using fixed assay. **a** Examples of seropositive immunoassays. Top: fixed CBA seropositive for NMDAR antibody IgG (CHR subject); human IgG is labelled green using a fluorescent secondary antibody. Bottom: immunohistochemistry showing IgG binding to fixed and permeabilised rat hippocampus (CHR subject seronegative for other specific antigenic targets); human IgG is labelled green using a fluorescent secondary antibody. **b** Confocal microscope image of IgG from CHR subject showing strong binding to cultured hippocampal neurons. Cell nuclei are DAPI-labelled; human IgG is labelled green using a fluorescent secondary antibody (AlexaFluor 488 mouse anti-human IgG1; Invitrogen) and hippocampal neurons are labelled red using mouse MAP2 antibodies (Monoclonal-Anti-MAP2, clone HM-2, Sigma Aldrich). Scalebar = 25 µm. **c** Distribution of antigen target by group. **d** Distribution of antibody isotype by group. **e** Distribution of antibody titre by group and assay type (any antibody or NMDAR antibody only); IgG = green, IgA = red, IgM = blue.

Using commercial, fixed assays, NSAbs were detected in 21 (8.3%) CHR subjects and 6 (5.2%) of matched, healthy controls (OR = 1.50; 95% CI: 0.58–3.90; *p* = 0.401). Prevalence did not differ by site (*p* = 0.800). IgG was the most commonly detected antibody isotype (48.1%) followed by IgA (33.3%) and IgM (22.2%). Isotype distribution did not differ between CHRs and HCs (IgG: *p* = 1.000; IgA: *p* = 0.283; IgM: *p* = 0.670). See Fig. 1c for distribution of all NSAb targets. NMDAR was the most commonly detected antigen in the CHR group (*n* = 11 [4.3%]; comprising IgG = 1 [0.4%], IgA = 6 [2.4%] and IgM = 4 [1.6%]) and in the HC group (*n* = 2 [1.7%]; comprising IgG = 1 [0.9%], IgA = 0 and IgM = 1 [0.9%]) (Fig. 1d). There was no difference in overall NMDAR antibody seroprevalence between the CHR and HC groups (*p* = 0.36). Although NMDAR antibody isotype distribution was not different between the two groups (IgG: *p* = 0.529; IgA: *p* = 0.183; IgM: *p* = 1.000), IgA NMDAR antibody seropositivity was seen in six CHR subjects but not in any HC.

On fixed CBAs, titres to specific antigens ranged from 1:10 to 1:320 in CHR subjects and from 1:32 to 1:320 in HCs (Fig. 1e), but none of these bound brain tissue slices on immunohistochemistry conducted in the same laboratory. However, 11 of 12 (91.7%) NSAb IgG seropositive samples bound cultured rat hippocampal neurons, with three samples showing strong binding. Serum from seven subjects (five CHR subjects, two HCs) had IgG, IgA or IgM antibodies which bound cerebellum or hippocampus sections (titre 1:32 to 1:320) without antigen-specific antibodies detected. No subjects were seropositive for more than one antigen on fixed CBA.

**Fig. 2:**
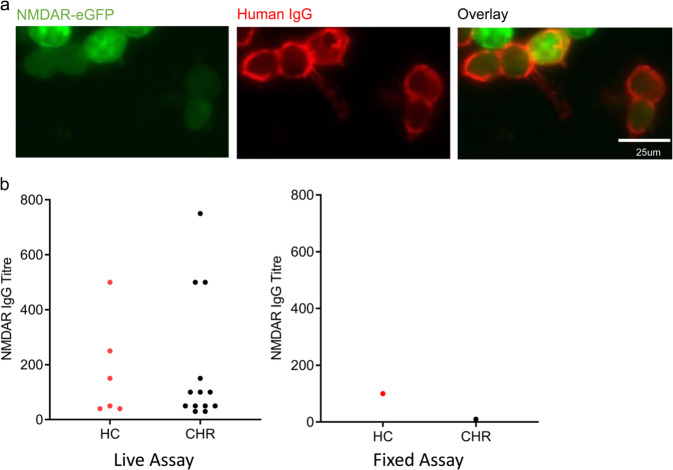
NMDAR IgG seroprevalence and associations of seropositivity—antibodies detected using live assay. **a** Representative example of NMDAR IgG-positive live CBA. Human IgG is labelled red using a fluorescent secondary antibody (AlexaFluor 568 goat anti-mouse IgG (H + L); Invitrogen) and shown binding to cells that co-express eGFP (green). Scalebar = 25 µm. **b** Distribution of NMDAR IgG titre by group and assay type (fixed vs. live assay).

Since studies that have used live CBA to detect NMDAR IgG in psychotic patients and controls have tended to yield greater seropositivity rates [23] and find case–control differences [10, 11, 22], indicating greater analytical sensitivity, we retested all samples for NMDAR IgG using a live CBA (Fig. 2a). Live CBA detected a significantly higher prevalence of NMDAR IgG antibodies in the total cohort than did fixed CBA (19 (5.1%) IgG seropositive subjects on live CBA vs. 2 (0.5%)) on fixed CBA: *p* = 0.0001), but were found similarly in 5.1% of CHR cases and 5.2% of HCs (*p* = 1.000). Prevalence did not differ by site (*p* = 0.145). Titres ranged from 1:30 to 1:750 in CHR subjects and from 1:40 to 1:500 in HCs (Fig. 2b). The two subjects who were seropositive for NMDAR IgG on fixed CBA were also seropositive on live CBA.

Seventeen of 19 samples that were NMDAR IgG seropositive on live assays were tested for binding to cultured rat hippocampal neurons: 10 of 17 (58.8%) samples bound neurons, with no samples showing strong binding. All four samples with a CBA titre of 1:250 or over bound neurons; of the 13 samples with lower titres, six (46.2%) bound neurons. IgG titre on CBA and hippocampal binding intensity score were positively correlated (*r* = 0.491; *p* = 0.048).

There were no age, sex, ethnicity or smoking status differences between seropositive and seronegative subjects (Table 2). S100B and hsCRP levels did not differ between seropositive and seronegative CHR subjects, for any NSAb or for NMDAR antibodies measured using either assay (Table 2).

**Table 2:** Demographic/basic clinical information by NSAb serostatus.

	Any antibody negative (*n* = 343)	Any antibody positive (fixed CBA) (*n* = 27)	*p*	NMDAR antibody negative (fixed CBA) (*n* = 357)	NMDAR antibody positive (fixed CBA) (*n* = 13)	*p*	NMDAR antibody negative (live CBA) (*n* = 351)	NMDAR antibody positive (live CBA) (*n* = 19)	*p*
Age	23.01 ± 4.60	22.33 ± 4.13	0.458	22.97 ± 4.57	22.69 ± 4.57	0.829	22.92 ± 4.50	23.79 ± 5.73	0.418
Sex [M: *n* (%)]	178 (51.9)	18 (66.7)	0.163	187 (52.4)	9 (69.2)	0.270	188 (53.6)	11 (57.9)	0.330
Ethnicity [*n* (%)]	White 231 (67.5), black 45 (13.2), other 66 (19.3)	White 23 (85.2), black 1 (3.7), other 3 (11.1)	0.148	White 44 (68.5), black 45 (12.6), other 67 (18.8)	White 10 (76.9), black 1 (7.7), other 2 (15.4)	0.797	White 243 (69.4), black 43 (12.3), other 64 (18.3)	White 11 (57.9), black 3 (15.8), other 5 (26.3)	0.563
Smoker^a^ [*n* (%)]	155 (45.9)	9 (33.3)	0.233	160 (45.5)	4 (30.8)	0.398	155 (44.8)	9 (47.4)	0.826
BMI	24.17 ± 4.98	23.19 ± 5.55	0.370	24.07	24.94	0.591	24.12 ± 5.02	23.82 ± 5.10	0.815
Current antipsychotic use [*n* (%)]	21 (6.1)	3 (11.1)	0.403	22 (6.2)	2 (15.4)	0.203	22 (6.3)	2 (10.5)	0.463
CRP (mg/L)^b^	1.14 ± 1.04	1.12 ± 1.21	0.991	1.14 ± 1.04	1.28 ± 1.41	0.925	1.14 ± 1.05	1.17 ± 0.94	0.506
S100B (ug/L)^b^	0.043 ± 0.058	0.038 ± 0.033	0.613	0.043 ± 0.057	0.04 ± 0.043	0.994	0.043 ± 0.058	0.037 ± 0.031	0.783

*CBA* cell-based assay, *S100B* S100 calcium-binding protein B, *CRP* C-reactive protein, *NMDAR* N-methyl-d-aspartate receptor, *BMI* body mass index.

^a^Data available for 365 of 370 subjects.

^b^Statistic adjusted for BMI, gender, age, ethnicity and smoking status.

There were no associations between antibody serostatus and self-reported medical history including neurological disorder, autoimmune disorder, head trauma, cancer, history of surgical procedures, CNS infection or serious infection (Supplementary Table 1). Notably the only subject that had had a CNS infection (meningitis) was seropositive for NMDAR IgG on live assay.

### Clinical outcomes of CHR subjects

Of the 254 CHR subjects from whom serum had been collected and tested for NSAbs, 49 (19.3%) developed a psychotic illness in the follow-up period. The subjects who transitioned to psychosis did not differ from those who did not transition in age or gender (Supplementary Table 2).

Of the subjects who transitioned to psychosis, 1 (2.0%) was seropositive for NMDAR antibodies on fixed CBA and 3 (6.1%) were seropositive for NMDAR IgG on live assay at baseline; of 205 CHR subjects who did not transition to psychosis, 10 (4.9%) were seropositive on fixed assay and 10 (4.9%) were seropositive on live assay (Supplementary Table 3). In logistic regression analyses, NMDAR antibody serostatus at baseline did not predict later transition to psychosis or nonremission from the CHR state, regardless of the assay used (fixed or live CBA) for antibody detection (Supplementary Tables 4–7).

Scores of functioning were assessed in CHR subjects at the assessment timepoints closest to two years after baseline. Mean change in GAF scores was negative (indicating worsening function) for seropositive subjects (live assay), and positive (indicating improvement) in seronegative subjects; in multiple regression analyses NMDAR antibody seropositivity was significantly associated with change in GAF disability scores (Supplementary Table 8). There was a significant negative correlation between NMDAR IgG titre (live assay) and change in GAF disability scores (Spearman’s rho = −0.542; *p* = 0.024), with higher titre subjects showing a more negative change in GAF scores, indicating deteriorating function.

### Cognitive associations of NMDAR antibody seropositivity in CHR subjects

Total RAVLT immediate recall score was lower in CHR subjects seropositive for NMDAR antibodies on fixed CBA compared to seronegative CHR subjects (*p* = 0.030). These differences became more substantial after controlling for total CAARMS score (*p* = 0.007), suggesting that the relationship with auditory verbal memory was not simply secondary to an effect of the severity of psychopathology (Table 3).

**Table 3:** Cognitive function and psychopathology scores by NMDAR antibody serostatus (descriptive means shown with *F* and *p* values from ANOVA).

	Antibody positive	Antibody negative	*F* (serostatus)	*p* (serostatus)
NMDAR antibody (IgG, IgA, IgM; fixed assay)				
AVLT IR total (7:160)	52.00 ± 13.79	63.06 ± 11.90	4.817	**0.030**
AVLT IR total (CAARMS adjusted) (5:127)	45.80 ± 10.69	62.50 ± 11.90	7.651	**0.007**
WAIS estimated total IQ (9:222)	84.22 ± 20.35	98.71 ± 16.90	6.687	**0.010**
WAIS estimated total IQ (CAARMS adjusted) (7:182)	83.29 ± 21.62	100.32 ± 16.86	7.134	**0.008**
CAARMS total (9:198)	49.00 ± 16.17	49.49 ± 15.00	0.001	0.981
BPRS-positive sx (11:224)	14.91 ± 5.43	13.55 ± 4.54	0.731	0.393
SANS (9:210)	20.44 ± 15.30	15.53 ± 11.74	1.074	0.301
YMRS (11:225)	4.27 ± 5.35	4.00 ± 4.79	0.03	0.957
MADRS (11:230)	20.91 ± 12.98	18.69 ± 9.16	0.650	0.421
NMDAR antibody (IgG; LIVE ASSAY)				
AVLT IR total (9:158)	58.00 ± 13.90	62.86 ± 12.03	0.929	0.337
AVLT IR total (CAARMS adjusted) (9:123)	58.00 ± 13.90	62.15 ± 12.13	0.826	0.365
WAIS estimated total IQ (12:219)	95.00 ± 19.98	98.32 ± 17.10	0.067	0.796
WAIS estimated total IQ (CAARMS adjusted) (12:177)	95.00 ± 19.98	100.01 ± 17.11	0.353	0.553
CAARMS total (13:194)	51.00 ± 11.90	49.37 ± 15.21	0.197	0.657
BPRS-positive sx (13:222)	14.62 ± 4.82	13.56 ± 4.57	0.683	0.409
SANS (13:206)	15.31 ± 11.39	15.76 ± 11.96	0.058	0.810
YMRS (12:224)	6.50 ± 5.16	3.89 ± 4.76	3.179	0.076
MADRS (13:228)	23.69 ± 9.05	18.51 ± 9.30	3.847	0.051

Bold values indicate statistical significance *p* < 0.05.

Numbers in brackets under variable names are the number of seropositive subjects and seronegative subjects for whom data were available for that variable. BPRS total score excluded from analysis due to heteroscedasticity.

*NMDAR* N-methyl-d-aspartate receptor, *AVLT* Rey Auditory Verbal Learning Task, *IR* immediate recall, *CAARMS* Comprehensive Assessment of At-Risk Mental States, *WAIS* Wechsler Adult Intelligence Scale III, *BPRS* Brief Psychiatric Rating Scale, *SANS* Scale for the Assessment of Negative Symptoms, *YMRS* Young Mania Rating Scale, *MADRS* Montgomery-Asberg Depression Rating Scale.

In order to explore whether the association with cognitive impairment was specific to verbal memory function, we then compared IQ (estimated using the WAIS-III) in seropositive and seronegative subjects. Mean WAIS-III IQ in NMDAR antibody seropositive CHR subjects was almost 15 points lower than that in seronegative CHR subjects (*p* = 0.010). This group difference also remained significant after controlling for total CAARMS score (*p* = 0.008) (Table 3). Both effects appeared to be driven by IgA/M antibodies: after restricting the analysis to IgA/M seropositive subjects only, both AVLT immediate recall (*p* = 0.011) and WAIS-III estimated IQ (*p* = 0.018) remained significantly impaired in seropositive subjects.

In fact, within the total CHR sample (*n* = 254), only 9 subjects had an IQ below 70 (the threshold for a diagnosis of intellectual disability) but 3 of these CHR subjects (33%) were seropositive for NMDAR antibodies. Subjects who were NMDAR IgG seropositive on live CBA did not differ from seronegative subjects on measures of cognition including AVLT immediate recall (*p* = 0.337) and IQ (*p* = 0.796).

### Symptomatic associations of NMDAR antibody seropositivity in CHR subjects

Nine of 13 (69.2%) subjects who were seropositive for NMDAR antibodies on live assay met DSM-IV criteria for current depressive episode, compared to 31.2% of seronegative subjects (*p* = 0.012). Rates of other DSM-IV diagnoses did not differ according to serostatus. There was no difference in the rates of specific DSM-IV diagnoses between NMDAR antibody fixed CBA seropositive and seronegative subjects (Supplementary Table 9).

While symptom severity scores were not significantly different between seropositive and seronegative subjects for most symptom domains when looking at antibodies detected using either assay, MADRS scores were borderline significantly higher in CHR subjects seropositive on live CBA (*p* = 0.051), indicating greater depression symptom severity. Across the whole CHR cohort, MADRS scores were weakly correlated with NMDAR IgG (live CBA) titre (Spearman’s rho = 0.167; *p* = 0.009).

There was no association between NMDAR antibody serostatus and CHR subtype (vulnerability group, attenuated psychosis symptoms [APS] or brief limited intermittent psychotic symptoms [BLIPS]) (fixed CBA: *p* = 0.316; live CBA: *p* = 0.865), although the numbers of subjects in the vulnerability (*n* = 41) and BLIPS (*n* = 16) subgroups were small.

### Association between blood–brain barrier disruption and symptoms

In order to assess the possible role of blood–brain barrier disruption as a moderating variable for the associations of NMDAR serostatus with symptom measures, we performed a preliminary (due to small n in the seropositive groups) analysis of the association between serum S100B levels and symptom scores in NMDAR antibody seropositive and seronegative groups. In subjects for whom NMDAR IgG were detected using live CBA, positive associations between S100B and symptom severity at baseline were observed for total BPRS score and for negative symptoms. In the seronegative group, modest negative associations between S100B and symptom severity were observed for multiple symptom scales (Supplementary Table 10).

### Longitudinal NSAb serostatus

One-hundred seventy-nine follow-up serum samples were available on 120 CHR subjects (modal interval between baseline and repeat blood sample: 12 months). Baseline serostatus was predictive of follow-up serostatus, i.e., subjects who were seropositive at baseline were more likely than subjects who were seronegative at baseline to be seropositive at follow-up. In total, of 109 CHR subjects who were seronegative at baseline and who had blood taken at subsequent visits, 9 (8.3%) were seropositive at subsequent visits (i.e., had new neuronal antibodies detectable). Of 11 subjects who were seropositive at baseline, 4 (36.4%) were still seropositive on at least one subsequent visit (*p* = 0.018). In 3 of these 4 subjects (75%), NSAbs detectable at follow-up were to the same antigen and of the same isotype.

None of the four subjects with persistent antibodies (i.e., NSAbs detectable at baseline and at a subsequent timepoint) became psychotic during follow-up. Only one of 9 subjects who developed *de novo* NSAbs at follow-up became psychotic: this subject developed NMDAR IgM antibodies (1:10) a year after initial assessment, having been seronegative at baseline; he went on to transition to psychosis a year later, but no blood samples were available at the time he became psychotic or afterwards.

Of five subjects who had NMDAR antibodies detectable at baseline and on whom follow-up samples were available, two (40%) had NSAbs at one or more follow-up point. One case with NMDAR IgA antibodies (1:32) at baseline had IgG antibodies at visit 2 (12 months) that bound hippocampus and cerebellum (1:100) but not NMDAR-specific antibodies detectable, and at visit 4 (24 months) had no antibodies at all detectable. One case with NMDAR IgM antibodies at baseline (1:100) had NMDAR IgM antibodies (1:100) detectable at visit 3. Neither of these cases developed psychosis.

Of 11 CHR subjects on whom serum was taken at the time of transition to psychosis, none were seropositive.

### Limbic volumes in NSAb seropositive CHR subjects

As only a small number of the CHR subjects who were NMDAR antibody seropositive had also had an MRI scan (*n* = 5), we investigated the relationship between autoantibodies and MRI measures in subjects who were seropositive for any NSAb (*n* = 11). CHR subjects who were seropositive for NSAbs had larger amgydala volumes (*p* = 0.048) than seronegative CHR subjects (*n* = 148). There were no group differences in hippocampal volume (Supplementary Table 11).

## Discussion

### Summary of results

This is the first study of CNS autoantibodies in people at CHR for psychosis. We found an overall seroprevalence of 8.3%, indicating that NSAbs are detectable in a minority of these individuals. The NMDAR was the most common target antigen and NMDAR IgGs were more sensitively detected with live versus fixed CBAs. The prevalence was not significantly greater than in HCs, mirroring findings from recent studies that have found similar case–control seroprevalence rates in patients with psychosis and other psychiatric and non-psychiatric disorders [1, 21, 53]. NMDAR antibody seropositivity was not associated with the development of a psychotic disorder, which was our main outcome of interest, but was associated with a deterioration in disability-associated functional status. Within the CHR sample, NMDAR antibody seropositivity was associated with impaired verbal memory performance, lower IQ and with depressive symptoms. Exploratory MRI analysis indicated that NSAb seropositivity was associated with increased amygdala volume.

### Relevance of NSAbs in CHR subjects

The combination of impaired cognitive function, severe affective psychopathology [46, 51, 54] and increased volume of limbic brain regions [55–58] bears some resemblance to (but is less severe than) the pattern of findings seen in the acute stages of many autoimmune encephalopathies. This suggests that NMDAR antibodies, and possibly other NSAbs, could play an analogous, but not necessarily identical, role in the pathophysiology of some patients who present with psychiatric symptoms [59]. This would be consistent with recent evidence that NMDAR antibodies isolated from psychiatric patients have pathogenicity in vitro and in vivo [9, 22, 24].

The reductions in verbal memory performance in NMDAR antibody-positive subjects are reminiscent of those seen in NMDAR encephalitis, and were of a comparable magnitude [19]. It is notable, however, that seropositive CHR subjects did not show other features of autoimmune encephalopathies, such as seizures or frank movement disorders. Neurological soft signs were not assessed in the present study, but subtle movement signs have been observed in a proportion of CHR subjects [60–62] and have been associated with NMDAR antibody seropositivity in patients with psychosis [9].

Furthermore, NMDAR antibody encephalitis is classically associated with a polymorphic affective psychosis with catatonic features [7], which is broader than the phenotypic associations of seropositivity that we observed. However a rodent model involving the intraventricular administration of NMDAR antibodies generated a cognitively impaired, anxious and depressive phenotype [63], which is consistent with our findings of an association between NMDAR antibodies and MADRS scores and current depressive episodes in CHR subjects. It has been suggested that the multifaceted symptomatology of NMDAR encephalitis represents more than the effects of the NMDAR antibody at the synapse, and that additional immune and non-immune factors help shape clinical presentation. Most passive immunisation animal models do not recapitulate a full encephalopathic phenotype, instead showing convergence around a more restricted set of symptoms, which could be conceived as a ‘ketamine-like’ synaptopathy [64], without features of frank CNS inflammation. If NMDAR antibodies do have a causal role in some psychiatric disorders, it is therefore possible that the effects represent more of a synaptopathic than a frank encephalitic process.

Additionally, the possibility that NMDAR antibodies could play a causal role in some CHR cases does not imply that the overall underlying immunology in these cases would be the same as in NMDAR encephalitis, a disorder which is characterised by the presence of NR1-specific intrathecal B cells. Indeed the importance of BBB disruption in determining the relevance of peripheral NMDAR antibodies in many human and animal studies of NSAb-mediated psychiatric disorders [9, 27, 64]) suggests that intrathecal synthesis may be minimal or absent in seropositive subjects with isolated psychiatric presentations.

Given the associations we report between NSAbs and both amygdala enlargement and affective symptomatology, it is interesting to note that whereas amygdala volumes are reduced in schizophrenia and first episode schizophrenic psychosis, they are increased in the first episode of affective psychoses and other nonschizophrenic psychoses (including brief psychotic disorder) [65]. The increase in amygdala volumes in our NSAb seropositive subjects could represent acute inflammation, as is the case in acute limbic encephalitis. Importantly, in the post-acute phase of autoimmune encephalitis there is evidence of volume loss in these same regions, which is in turn linked to the severity of persisting deficits [5, 15, 17]. If the neuroanatomical associations of NSAb seropositivity truly represent a similar mechanism to that of autoimmune encephalitis, one testable hypothesis is that these brain regions will show accelerated atrophy or volume loss at follow-up scans in seropositive CHR subjects.

Prüss, Finke and colleagues have highlighted associations between IgA NMDAR antibodies and cognitive impairments in multiple clinical contexts [2, 18, 66]. In the present study, 6 CHR subjects had IgA NMDAR antibodies, compared with no HCs. It is thus possible that IgA NMDAR antibodies drive the cognitive impairment observed in our seropositive subjects. IgM, and many IgAs, are not frequently reported in the CSF, although this may potentially reflect a bias towards the measurement of IgG only. While IgA can exist as monomers in blood, IgMs are multimerised antibodies, potentially making their crossing of the blood–brain barrier less likely than IgGs.

We observed that in CHR individuals NMDAR antibody serostatus fluctuates over time. Pan and colleagues recently reported similar findings in animals and human subjects (following stroke): a majority of individuals remained seronegative, and a minority either remained seropositive or acquired de novo antibodies at 1–3-year follow-up [67]. What causes these fluctuations, and whether they have any direct clinical consequences, has not been established (although there is some emerging evidence for chronic stress as a potential inducer of NMDAR antibodies).

### Comparison of cell-based assays

In the largest direct assay comparison reported to date, we found that live CBA detects a nine times higher prevalence of IgG NMDAR antibodies in serum than fixed CBA, confirming a previous report in a first episode psychosis cohort [23]. In this earlier report, even in the absence of seropositivity on fixed CBA, these IgG both targeted and were capable of altering the surface dynamics of the NMDAR, suggesting pathogenic potential.

In total 72.4% of IgG seropositive samples bound hippocampal neurons; this was more true of IgG seropositive samples on fixed CBA (91.7%) than on live CBA (58.8%). This is consistent with live CBA potentially detecting antibodies with higher sensitivity than the fixed CBA. The fact that in our laboratory not all live CBA-positive samples bound rat hippocampal neurons (see also ref. [21]) does not undermine the potential disease-relevance of NMDAR IgG seropositivity, particularly as these antibodies have pathogenic potential at the level of the NMDAR [22, 23]. Immunocytochemistry with cultured neurons, as with all assays, has a detection threshold, and this should be understood in the context that CBAs overexpress the antigen at the surface but in immunocytochemistry expression levels are broadly physiological. Therefore, it might be expected that some lower titre samples that are positive on live assay will not demonstrate binding to neurons. The observed correlation between NMDAR IgG live CBA titre and hippocampal binding intensity is suggestive that live CBA is measuring genuine IgG binding, and indeed the live CBA threshold above which NMDAR IgG-positive samples will reliably bind to neurons appears to be 1:250.

### The role of the blood–brain barrier

Ehrenreich and colleagues have stressed that for peripheral NSAbs to be clinically relevant there may also need to be disruption of the BBB [64]. This has been demonstrated in mice with an ApoE-/- haplotype [68] and in humans with a ‘history of neurotrauma’ or obstetric complications as medical proxies [9]. A parsimonious interpretation is that when the integrity of the BBB is compromised, NSAbs have greater access to the CNS and are more likely to disrupt brain function [28]. However, it is also possible that serum NSAbs are peripheral biomarkers of a central dysfunction that is accompanied by BBB disruption, such as neuroinflammation. Consistent with the role of the BBB as proposed by Ehrenreich and colleagues, in a supplemental analysis we observed positive associations between S100B levels (a widely used proxy for BBB disruption [28, 31]) and symptom scores in subjects who were seropositive for NMDAR antibodies on live CBA; these associations were in the opposite direction in subjects without detectable serum antibodies. Given the small numbers of seropositive subjects, however, this finding mandates replication in a larger sample, potentially with an additional marker of BBB disruption. The ‘gold standard’ of BBB quantification in humans is the CSF-serum albumin quotient, which has consistently been found to be abnormal in psychosis [28]. However, this requires a lumbar puncture, which is not usually part of the clinical assessment of patients with psychosis, let alone individuals at CHR [69]. A less invasive method for BBB quantification in these individuals is the use of dynamic contrast-enhanced MRI [70].

### Antibodies as biomarkers in the CHR state

Separately, or in addition to, the question of the pathogenicity of NMDAR antibodies in the CHR state, the present study indicates a potential role for these antibodies as biomarkers of poor cognition, depressive symptomatology and/or outcome in the CHR state.

In CHR subjects, severe psychopathology and cognitive impairments, and alterations in limbic brain volumes at presentation are associated with an increased risk of later transition to psychosis [12–14, 71, 72]. Impaired verbal memory performance in CHR individuals is one of the strongest cognitive predictors of subsequent transition to psychosis [73, 74]. Low IQ has also been identified as a predictor of conversion to psychosis in CHR subjects [75], and is an established risk factor for psychosis in the general population [76]. Our study suggests, complementing similar findings in other patient groups [2, 18, 20, 66], that NMDAR antibodies may have a role as a biomarker of cognitive impairment in CHR subjects. Since we observed that antibody serostatus fluctuates over time, an important question is whether cognitive status fluctuates in association with serostatus. This latter might be expected if NMDAR antibodies represent state (rather than trait) biomarkers for poor cognition: indeed most passive transfer models suggest that the antibody effects are reversible. However a different relationship might be expected if serostatus is in fact a marker of poor cognitive performance due to the impact of an as-yet undetermined confounder.

In our study, NMDAR antibodies did not predict transition to psychosis, as defined by operationalised criteria, or nonremission from the CHR state, despite all but one NMDAR IgG seropositive subjects still meeting CHR criteria at follow-up. NMDAR antibody seropositivity was however associated with a deterioration in disability-associated functioning, short of transition, and indeed greater antibody titre was associated with greater deterioration in function, overall suggesting that NMDAR antibody serostatus should be further evaluated as a predictive marker of functional outcome.

### Limitations

We measured NSAbs in serum. Assessing NSAbs in CSF may provide a better way of investigating their role in some autoimmune CNS disorders, but lumbar puncture is more invasive and is not usually carried out in CHR subjects. However, while CSF NSAbs are important for the diagnosis of autoimmune encephalitis, this does not imply that serum NSAbs are irrelevant in psychiatric conditions such as the CHR state. As Castillo-Gomez and colleagues have demonstrated, in conditions in which antibody production is putatively lower than in autoimmune encephalitis the brain may act as ‘immunoprecipitator’ of CSF NSAbs, and NSAbs may not be detectable in CSF, despite having functional consequences in the brain [68]. Although we studied a large sample of CHR subjects, because only a minority were antibody-positive, the numbers of subjects in some of the comparisons was small and hence we did not perform corrections for multiple comparisons. We also cannot exclude the possibility that we failed to detect some differences because of limited statistical power. This issue may be addressed by pooling data from different CHR cohorts, as in the Harmony collaboration (https://www.pronia.eu/news-pronia/news-detail/tx_news/initiation-of-the-multi-center-collaboration-harmony-in-october-copy-1/).

## Supplementary information

Supplementary Information

## References

[1] Dahm L, Ott C, Steiner J, Stepniak B, Teegen B, Saschenbrecker S (2014). Seroprevalence of autoantibodies against brain antigens in health and disease. Ann Neurol.

[2] Doss S, Wandinger KP, Hyman BT, Panzer JA, Synofzik M, Dickerson B (2014). High prevalence of NMDA receptor IgA/IgM antibodies in different dementia types. Ann Clin Transl Neurol.

[3] Zandi MS, Irani SR, Lang B, Waters P, Jones PB, McKenna P (2011). Disease-relevant autoantibodies in first episode schizophrenia. J Neurol.

[4] Pollak TA, Beck K, Irani SR, Howes OD, David AS, McGuire PK (2016). Autoantibodies to central nervous system neuronal surface antigens: psychiatric symptoms and psychopharmacological implications. Psychopharmacology..

[5] Heine J, Pruss H, Bartsch T, Ploner CJ, Paul F, Finke C (2015). Imaging of autoimmune encephalitis-Relevance for clinical practice and hippocampal function. Neuroscience..

[6] Bien CG, Vincent A, Barnett MH, Becker AJ, Blumcke I, Graus F (2012). Immunopathology of autoantibody-associated encephalitides: clues for pathogenesis. Brain: a J Neurol.

[7] Al-Diwani A, Handel A, Townsend L, Pollak T, Leite MI, Harrison PJ (2019). The psychopathology of NMDAR-antibody encephalitis in adults: a systematic review and phenotypic analysis of individual patient data. Lancet Psychiatry..

[8] Dalmau J, Gleichman AJ, Hughes EG, Rossi JE, Peng X, Lai M (2008). Anti-NMDA-receptor encephalitis: case series and analysis of the effects of antibodies. Lancet Neurol.

[9] Hammer C, Stepniak B, Schneider A, Papiol S, Tantra M, Begemann M (2014). Neuropsychiatric disease relevance of circulating anti-NMDA receptor autoantibodies depends on blood-brain barrier integrity. Mol Psychiatry.

[10] Lennox BR, Palmer-Cooper EC, Pollak T, Hainsworth J, Marks J, Jacobson L (2017). Prevalence and clinical characteristics of serum neuronal cell surface antibodies in first-episode psychosis: a case-control study. Lancet Psychiatry.

[11] Pathmanandavel K, Starling J, Merheb V, Ramanathan S, Sinmaz N, Dale RC (2015). Antibodies to surface dopamine-2 receptor and N-methyl-D-aspartate receptor in the first episode of acute psychosis in children. Biol Psychiatry.

[12] Fusar-Poli P, Deste G, Smieskova R, Barlati S, Yung AR, Howes O (2012). Cognitive functioning in prodromal psychosis: a meta-analysis. Arch Gen Psychiatry.

[13] Perkins DO, Jeffries CD, Cornblatt BA, Woods SW, Addington J, Bearden CE (2015). Severity of thought disorder predicts psychosis in persons at clinical high-risk. Schizophrenia Res.

[14] Mechelli A, Riecher-Rossler A, Meisenzahl EM, Tognin S, Wood SJ, Borgwardt SJ (2011). Neuroanatomical abnormalities that predate the onset of psychosis: a multicenter study. Arch Gen Psychiatry.

[15] Finke C, Kopp UA, Pajkert A, Behrens JR, Leypoldt F, Wuerfel JT (2016). Structural hippocampal damage following anti-N-methyl-D-aspartate receptor encephalitis. Biol Psychiatry.

[16] Finke C, Kopp UA, Pruss H, Dalmau J, Wandinger KP, Ploner CJ (2012). Cognitive deficits following anti-NMDA receptor encephalitis. J Neurol Neurosurg Psychiatry.

[17] Finke C, Kopp UA, Scheel M, Pech LM, Soemmer C, Schlichting J (2013). Functional and structural brain changes in anti-N-methyl-D-aspartate receptor encephalitis. Ann Neurol.

[18] Bartels F, Stronisch T, Farmer K, Rentzsch K, Kiecker F, Finke C (2019). Neuronal autoantibodies associated with cognitive impairment in melanoma patients. Ann Oncol: Off J Eur Soc Med Oncol/ESMO.

[19] Finke C, Bartels F, Lutt A, Pruss H, Harms L (2017). High prevalence of neuronal surface autoantibodies associated with cognitive deficits in cancer patients. J Neurol.

[20] Westman G, Studahl M, Ahlm C, Eriksson BM, Persson B, Ronnelid J (2016). N-methyl-d-aspartate receptor autoimmunity affects cognitive performance in herpes simplex encephalitis. Clin Microbiol Infect.

[21] Gaughran F, Lally J, Beck K, McCormack R, Gardner-Sood P, Coutinho E (2017). Brain-relevant antibodies in first-episode psychosis: a matched case-control study. Psychol Med.

[22] Jezequel J, Johansson EM, Dupuis JP, Rogemond V, Grea H, Kellermayer B (2017). Dynamic disorganization of synaptic NMDA receptors triggered by autoantibodies from psychotic patients. Nat Commun.

[23] Jezequel J, Rogemond V, Pollak T, Lepleux M, Jacobson L, Grea H (2017). Cell- and single molecule-based methods to detect anti-N-methyl-D-aspartate receptor autoantibodies in patients with first-episode psychosis from the OPTiMiSE project. Biol Psychiatry.

[24] Castillo-Gomez E, Oliveira B, Tapken B, Bertrand S, Klein-Schmidt C, Pan H (2016). All naturally occurring autoantibodies against the NMDA receptor subunit NR1 have pathogenic potential irrespective of epitope and immunoglobulin class. Mol Psychiatry.

[25] Kreye J, Wenke NK, Chayka M, Leubner J, Murugan R, Maier N (2016). Human cerebrospinal fluid monoclonal N-methyl-D-aspartate receptor autoantibodies are sufficient for encephalitis pathogenesis. Brain: J Neurol.

[26] Makuch M, Wilson R, Al-Diwani A, Varley J, Kienzler AK, Taylor J (2018). N-methyl-D-aspartate receptor antibody production from germinal center reactions: therapeutic implications. Ann Neurol.

[27] Hammer C, Zerche M, Schneider A, Begemann M, Nave KA, Ehrenreich H (2014). Apolipoprotein E4 carrier status plus circulating anti-NMDAR1 autoantibodies: association with schizoaffective disorder. Mol Psychiatry.

[28] Pollak TA, Drndarski S, Stone JM, David AS, McGuire P, Abbott NJ (2017). The blood-brain barrier in psychosis. Lancet Psychiatry.

[29] Schumberg K, Polyakova M, Steiner J, Schroeter ML (2016). Serum S100B is related to illness duration and clinical symptoms in schizophrenia-A meta-regression analysis. Front Cell Neurosci.

[30] Aleksovska K, Leoncini E, Bonassi S, Cesario A, Boccia S, Frustaci A (2014). Systematic review and meta-analysis of circulating S100B blood levels in schizophrenia. PLoS ONE.

[31] Strathmann FG, Schulte S, Goerl K, Petron DJ (2014). Blood-based biomarkers for traumatic brain injury: evaluation of research approaches, available methods and potential utility from the clinician and clinical laboratory perspectives. Clin Biochem.

[32] Fusar-Poli P, Cappucciati M, Borgwardt S, Woods SW, Addington J, Nelson B (2016). Heterogeneity of psychosis risk within individuals at clinical high risk: a meta-analytical stratification. JAMA Psychiatry.

[33] Fusar-Poli P, Bonoldi I, Yung AR, Borgwardt S, Kempton MJ, Valmaggia L (2012). Predicting psychosis: meta-analysis of transition outcomes in individuals at high clinical risk. Arch Gen Psychiatry.

[34] McGuire P, Sato JR, Mechelli A, Jackowski A, Bressan RA, Zugman A (2015). Can neuroimaging be used to predict the onset of psychosis?. Lancet Psychiatry..

[35] Kraan TC, Velthorst E, Themmen M, Valmaggia L, Kempton MJ, McGuire P (2018). Child maltreatment and clinical outcome in individuals at ultra-high risk for psychosis in the EU-GEI high risk study. Schizophrenia Bull.

[36] Hatch SL, Frissa S, Verdecchia M, Stewart R, Fear NT, Reichenberg A (2011). Identifying socio-demographic and socioeconomic determinants of health inequalities in a diverse London community: the South East London Community Health (SELCoH) study. BMC Public Health.

[37] Bebbington P, Nayani T (1995). The psychosis screening questionnaire. Int J Methods Psychiatr Res.

[38] Yung AR, Yuen HP, McGorry PD, Phillips LJ, Kelly D, Dell’Olio M (2005). Mapping the onset of psychosis: the comprehensive assessment of at-risk mental states. Aust NZ J Psychiatry.

[39] Lukoff D, Liberman RP, Nuechterlein KH (1986). Symptom monitoring in the rehabilitation of schizophrenic patients. Schizophrenia Bull.

[40] Andreasen NC (1989). The scale for the assessment of negative symptoms (SANS): conceptual and theoretical foundations. Br J Psychiatry Suppl.

[41] Young RC, Biggs JT, Ziegler VE, Meyer DA (1978). A rating scale for mania: reliability, validity and sensitivity. Br J Psychiatry: J Ment Sci.

[42] Montgomery SA, Asberg M (1979). A new depression scale designed to be sensitive to change. Br J Psychiatry: J Ment Sci.

[43] Schmidt M (1996). Rey auditory and verbal learning test: a handbook.

[44] Wechsler D (1981). Manual for the Wechsler Adult Intelligence Scale—Revised.

[45] Wandinger KP, Saschenbrecker S, Stoecker W, Dalmau J (2011). Anti-NMDA-receptor encephalitis: a severe, multistage, treatable disorder presenting with psychosis. J Neuroimmunol.

[46] Irani SR, Bera K, Waters P, Zuliani L, Maxwell S, Zandi MS (2010). N-methyl-D-aspartate antibody encephalitis: temporal progression of clinical and paraclinical observations in a predominantly non-paraneoplastic disorder of both sexes. Brain: J Neurol.

[47] Irani SR, Alexander S, Waters P, Kleopa KA, Pettingill P, Zuliani L (2010). Antibodies to Kv1 potassium channel-complex proteins leucine-rich, glioma inactivated 1 protein and contactin-associated protein-2 in limbic encephalitis, Morvan’s syndrome and acquired neuromyotonia. Brain..

[48] Dickerson F, Stallings C, Origoni A, Vaughan C, Khushalani S, Yang S (2013). C-reactive protein is elevated in schizophrenia. Schizophrenia Res.

[49] Allen P, Chaddock CA, Egerton A, Howes OD, Bonoldi I, Zelaya F (2016). Resting hyperperfusion of the hippocampus, midbrain, and basal ganglia in people at high risk for psychosis. Am J Psychiatry.

[50] Witthaus H, Mendes U, Brune M, Ozgurdal S, Bohner G, Gudlowski Y (2010). Hippocampal subdivision and amygdalar volumes in patients in an at-risk mental state for schizophrenia. J Psychiatry Neurosci.

[51] Gibson LL, Pollak TA, Blackman G, Thornton M, Moran N, David AS (2019). The psychiatric phenotype of anti-NMDA receptor encephalitis. J Neuropsychiatry Clin Neurosci.

[52] Dingemans PM, Linszen DH, Lenior ME, Smeets RM (1995). Component structure of the expanded Brief Psychiatric Rating Scale (BPRS-E). Psychopharmacology..

[53] Steiner J, Teegen B, Schiltz K, Bernstein HG, Stoecker W, Bogerts B (2014). Prevalence of N-methyl-D-aspartate receptor autoantibodies in the peripheral blood: healthy control samples revisited. JAMA Psychiatry.

[54] Kayser MS, Titulaer MJ, Gresa-Arribas N, Dalmau J (2013). Frequency and characteristics of isolated psychiatric episodes in anti-N-methyl-d-aspartate receptor encephalitis. JAMA Neurol.

[55] Wagner J, Weber B, Elger CE (2015). Early and chronic gray matter volume changes in limbic encephalitis revealed by voxel-based morphometry. Epilepsia..

[56] Wagner J, Witt JA, Helmstaedter C, Malter MP, Weber B, Elger CE (2015). Automated volumetry of the mesiotemporal structures in antibody-associated limbic encephalitis. J Neurol Neurosurg Psychiatry.

[57] Urbach H, Soeder BM, Jeub M, Klockgether T, Meyer B, Bien CG (2006). Serial MRI of limbic encephalitis. Neuroradiology.

[58] Taniguchi G, Fuse H, Okamura Y, Mori H, Kondo S, Kasai K (2018). Improvement in anti-N-methyl-d-aspartate receptor antibody-mediated temporal lobe epilepsy with amygdala enlargement without immunotherapy. Epilepsy Behav Case Rep.

[59] Al-Diwani A, Pollak T, Langford A, Lennox B (2017). Synaptic and neuronal autoantibody-associated psychiatric syndromes (SNAps): Controversies and Hypotheses. Front Psychiatry.

[60] Dean DJ, Mittal VA (2015). Spontaneous parkinsonisms and striatal impairment in neuroleptic free youth at ultrahigh risk for psychosis. NPJ Schizophr.

[61] Dean DJ, Kent JS, Bernard JA, Orr JM, Gupta T, Pelletier-Baldelli A (2015). Increased postural sway predicts negative symptom progression in youth at ultrahigh risk for psychosis. Schizophrenia Res.

[62] Tamagni C, Studerus E, Gschwandtner U, Aston J, Borgwardt S, Riecher-Rossler A (2013). Are neurological soft signs pre-existing markers in individuals with an at-risk mental state for psychosis?. Psychiatry Res.

[63] Planaguma J, Leypoldt F, Mannara F, Gutierrez-Cuesta J, Martin-Garcia E, Aguilar E (2015). Human N-methyl D-aspartate receptor antibodies alter memory and behaviour in mice. Brain: J Neurol.

[64] Ehrenreich H (2017). Autoantibodies against the N-methyl-d-aspartate receptor subunit NR1: untangling apparent inconsistencies for clinical practice. Front Immunol.

[65] Velakoulis D, Wood SJ, Wong MT, McGorry PD, Yung A, Phillips L (2006). Hippocampal and amygdala volumes according to psychosis stage and diagnosis: a magnetic resonance imaging study of chronic schizophrenia, first-episode psychosis, and ultra-high-risk individuals. Arch Gen Psychiatry.

[66] Pruss H, Holtje M, Maier N, Gomez A, Buchert R, Harms L (2012). IgA NMDA receptor antibodies are markers of synaptic immunity in slow cognitive impairment. Neurology..

[67] Pan H, Steixner-Kumar AA, Seelbach A, Deutsch N, Ronnenberg A, Tapken D, et al. Multiple inducers and novel roles of autoantibodies against the obligatory NMDAR subunit NR1: a translational study from chronic life stress to brain injury. Mol Psychiatry. 2020. [Epub ahead of print]. 10.1038/s41380-020-0672-1 PMC8440197 32089545

[68] Castillo-Gomez E, Kastner A, Steiner J, Schneider A, Hettling B, Poggi G (2016). The brain as immunoprecipitator of serum autoantibodies against N-Methyl-D-aspartate receptor subunit NR1. Ann Neurol.

[69] Pollak TA, Lennox BR (2018). Time for a change of practice: the real-world value of testing for neuronal autoantibodies in acute first-episode psychosis. BJPsych Open.

[70] Heye AK, Culling RD, Valdes Hernandez Mdel C, Thrippleton MJ, Wardlaw JM (2014). Assessment of blood-brain barrier disruption using dynamic contrast-enhanced MRI. A systematic review. Neuroimage Clin.

[71] Lieberman JA, Girgis RR, Brucato G, Moore H, Provenzano F, Kegeles L (2018). Hippocampal dysfunction in the pathophysiology of schizophrenia: a selective review and hypothesis for early detection and intervention. Mol Psychiatry.

[72] Phillips LJ, Velakoulis D, Pantelis C, Wood S, Yuen HP, Yung AR (2002). Non-reduction in hippocampal volume is associated with higher risk of psychosis. Schizophrenia Res.

[73] Simon AE, Gradel M, Cattapan-Ludewig K, Gruber K, Ballinari P, Roth B (2012). Cognitive functioning in at-risk mental states for psychosis and 2-year clinical outcome. Schizophrenia Res.

[74] Hauser M, Zhang JP, Sheridan EM, Burdick KE, Mogil R, Kane JM (2017). Neuropsychological test performance to enhance identification of subjects at clinical high risk for psychosis and to be most promising for predictive algorithms for conversion to psychosis: a meta-analysis. J Clin Psychiatry.

[75] Ziermans T, de Wit S, Schothorst P, Sprong M, van Engeland H, Kahn R (2014). Neurocognitive and clinical predictors of long-term outcome in adolescents at ultra-high risk for psychosis: a 6-year follow-up. PLoS ONE.

[76] Doody GA, Johnstone EC, Sanderson TL, Owens DG, Muir WJ (1998). ‘Pfropfschizophrenie’ revisited. Schizophrenia in people with mild learning disability. Br J Psychiatry: J Ment Sci.

