# Supplementary material for: Clinical, cognitive and neuroanatomical associations of serum NMDAR autoantibodies in people at clinical high risk for psychosis
Source: Mol Psychiatry. 2020 Oct 19;26(6):2590–604. doi: 10.1038/s41380-020-00899-w (PMC8440194; doi:10.1038/s41380-020-00899-w)
Supplement: Supplementary file 1 — Supplementary Information [file 41380_2020_899_MOESM1_ESM.docx]

Appendix 1: Substrates for fixed assays (Euroimmun AG, Lübeck, Germany)

1. Hippocampus rat

2. Cerebellum rat

3. Cerebellum monkey

4. Nerve monkey

5. Intestine monkey

6. Pancreas monkey

7. GAD65

8. NMDA-NR1

9. GABAB

10. AQP4

11. LGI1

12. CASPR2

13. GRM5=mGluR5

14. AMPA 1+2

15. Amphiphysin

16. CARPVIII

17. CV2

18. DNER

19. GAD67

20. GRM1 = mGluR1

21. Homer 3

22. Hu

23. ITPR1

24. Ma2

25. pre-GLRA1b (induced mixed substrate)

26. pre-GLRA1b (uninduced mixed substrate) (as negative control)

27. Recoverin

28. Rho GTPase activating protein 26 (ARHGAP26)

29. Ri

30. untransfected cells acetone fixed (as negative control)

31. untransfected cells formalin fixed (as negative control)

32. Yo

33. ZIC4

34. DPPX

35. IgLon5

| Medical category (data available on n subjects) | Antibody positive fixed CBA (n=10) | Antibody negative fixed CBA | p | Antibody positive live CBA (n=13) | Antibody negative live CBA | p |  |
| --- | --- | --- | --- | --- | --- | --- | --- |
| Neurological | 0 (0.0) | 15 (7.0) | 1.000 | 1 (7.7) | 14 (6.7) | 0.606 |  |
| Autoimmune | 0 (0.0) | 4 (1.9) | 1.000 | 1 (7.7) | 3 (1.4) | 0.214 |  |
| Allergic/atopic | 0 (0.0) | 5 (2.3) | 1.000 | 0 (0.0) | 5 (2.4) | 1.000 |  |
| Cancer | 0 (0.0) | 2 (0.9) | 1.000 | 0 (0.0) | 2 (1.0) | 1.000 |  |
| **Surgery** | 6 (60.0) | 80 (37.4) | 0.188 | 8 (61.5) | 78 (37.0) | 0.086 |  |
| Serious infection | 1 (10.0) | 17 (8.0) | 0.579 | 1 (7.7) | 17 (8.1) | 1.000 |  |
| Brain infection | 0 (0.0) | 1 (0.5) | 1.000 | 1 (7.7) | 0 (0.0) | 0.059 |  |
| Head injury | 0 (0.0) | 2 (0.9) | 1.000 | 0 (0.0) | 2 (1.0) | 1.000 |  |

Supplementary table 1: self-reported medical history by NMDAR antibody serostatus. Diagnoses were self-reported by subjects following one of four open-ended questions, with prompts to specify the relevant problems if answered affirmatively: ‘Have you seen your GP or a specialist in the last 2 years?’, ‘Have you had any operations?’, ‘Have you ever been seriously ill, or did you have to spend time in hospital?’, ‘Do you suffer from any physical problem? e.g asthma, heart disease, thyroid problems, diabetes, epilepsy, etc’.

|  | CHR-NT (n=205) | CHR-T (n=49) | p |
| --- | --- | --- | --- |
| Age | 22.74 ± 4.99 | 22.53 ± 5.11 | 0.796 |
| Sex [M: n (%)] | 107 (52.2) | 29 (59.2) | 0.427 |
| Ethnicity [n (%)] | White 147 (72.1), Black 14 (6.9), Other 43 (21.1) | White 31 (63.3), Black 8 (16.3), Other 10 (20.4) | 0.105 |
| Smoker?* [n (%)] | 113 (55.9) | 20 (42.6) | 0.097 |
| BMI | 24.33 | 24.22 | 0.910 |
| S100B (ug/L) | 0.04 ± 0.05 | 0.04 ± 0.05 | 0.826 |
| C-reactive protein (CRP) (mg/L) | 1.25 ± 1.11 | 1.15 ± 0.99 | 0.996 |

Supplementary table 2: demographic and basic clinical information in CHR subjects who did (CHR-T) and did not (CHR-NT) transition to psychosis

*data available for 249 of 254 subjects

Abbreviations: CHR: clinical high risk, CHR-T: clinical high risk – transition, CHR-NT: clinical high risk – nontransition, S100B: S100 calcium-binding protein B, CRP: C-reactive protein

|  | CHR-NT (n=205) | CHR-T (n=49) | p | CHR-R (n=187) | CHR-NR (n=67) | p |  |
| --- | --- | --- | --- | --- | --- | --- | --- |
| Any antibody fixed assay [n (%)] | 19 (9.3) | 2 (4.1) | 0.385 | 14 (7.5) | 7 (10.4) | 0.450 |  |
| NMDAR antibody fixed assay [n (%)] | 10 (4.9) | 1 (2.0) | 0.696 | 9 (4.8) | 2 (3.0) | 0.733 |  |
| NMDAR IgG fixed assay [n (%)] | 1 (0.5) | 0 (0.0) | 1.000 | 1 (0.5) | 0 (0.0) | 1.000 |  |
| NMDAR IgA fixed assay [n (%)] | 6 (2.9) | 0 (0.0) | 0.599 | 4 (2.1) | 2 (3.0) | 0.656 |  |
| NMDAR IgM fixed assay [n (%)] | 3 (1.5) | 1 (2.0) | 0.578 | 4 (2.1) | 0 (0.0) | 0.576 |  |
| NMDAR IgG live assay [n (%)] | 10 (4.9) | 3 (6.1) | 0.720 | 12 (6.4) | 1 (1.5) | 0.194 |  |

Supplementary table 3: NSAb serostatus in CHR subjects who did (CHR-T) and did not (CHR-NT) transition to psychosis and subjects who remitted (CHR-R) and did not remit (CHR-NR) from the CHR state

Abbreviations: CHR: clinical high risk, CHR-T: clinical high risk – transition, CHR-NT: clinical high risk – nontransition, CHR-R: clinical high risk – remission, CHR-NR: clinical high risk – nonremission, NMDAR: N-methyl-d-aspartate receptor.

|  | B | S.E. | Wald | Sig. | Exp(B) |
| --- | --- | --- | --- | --- | --- |
| Age | -0.005 | 0.035 | 0.021 | 0.884 | 0.995 |
| Gender | 0.388 | 0.347 | 1.254 | 0.263 | 1.475 |
| White ethnicity | -0.108 | 0.422 | 0.065 | 0.798 | 0.898 |
| Black ethnicity | 0.956 | 0.591 | 2.619 | 0.106 | 2.600 |
| NMDAR antibody serostatus (fixed assay) | 1.220 | 1.098 | 1.236 | 0.266 | 3.388 |

Supplementary table 4: Logistic regression illustrating association between NMDAR antibody serostatus (fixed assay) and transition to psychosis.

Abbreviations: NMDAR: N-methyl-d-aspartate receptor.

|  | B | S.E. | Wald | Sig. | Exp(B) |
| --- | --- | --- | --- | --- | --- |
| Age | -0.005 | 0.035 | 0.025 | 0.875 | 0.995 |
| Gender | 0.348 | 0.345 | 1.017 | 0.313 | 1.416 |
| White ethnicity | -0.147 | 0.420 | 0.122 | 0.727 | 0.864 |
| Black ethnicity | 0.924 | 0.586 | 2.490 | 0.115 | 2.520 |
| NMDAR IgG serostatus (live assay) | -0.194 | 0.717 | 0.074 | 0.786 | 0.823 |

Supplementary table 5: Logistic regression illustrating association between NMDAR IgG serostatus (live assay) and transition to psychosis.

Abbreviations: NMDAR: N-methyl-d-aspartate receptor.

|  | B | S.E. | Wald | Sig. | Exp(B) |
| --- | --- | --- | --- | --- | --- |
| Gender | 0.010 | 0.029 | 0.131 | 0.718 | 1.010 |
| Age | -0.078 | 0.293 | 0.071 | 0.791 | 0.925 |
| White ethnicity | 0.352 | 0.372 | 0.894 | 0.344 | 1.422 |
| Black ethnicity | 0.003 | 0.609 | 0.000 | 0.996 | 1.003 |
| NMDAR antibody serostatus (fixed assay) | 0.581 | 0.804 | 0.522 | 0.470 | 1.788 |

Supplementary table 6: Logistic regression illustrating association between NMDAR antibody serostatus (fixed assay) and remission from the CHR state.

Abbreviations: NMDAR: N-methyl-d-aspartate receptor.

|  | B | S.E. | Wald | Sig. | Exp(B) |
| --- | --- | --- | --- | --- | --- |
| Gender | 0.014 | 0.029 | 0.243 | 0.622 | 1.014 |
| Age | -0.127 | 0.295 | 0.187 | 0.665 | 0.880 |
| White ethnicity | 0.342 | 0.374 | 0.835 | 0.361 | 1.407 |
| Black ethnicity | 0.038 | 0.613 | 0.004 | 0.951 | 1.039 |
| NMDAR IgG serostatus (live assay) | 1.541 | 1.058 | 2.123 | 0.145 | 4.669 |

Supplementary table 7: Logistic regression illustrating association between NMDAR IgG serostatus (live assay) and remission from the CHR state.

Abbreviations: NMDAR: N-methyl-d-aspartate receptor.

|  | Antibody negative | Antibody positive | F (serostatus) | p (serostatus) |  |
| --- | --- | --- | --- | --- | --- |

| NMDAR ANTIBODY (IgG, IgA, IgM; FIXED ASSAY) |  |  |  |  |
| --- | --- | --- | --- | --- |
| Change in GAF symptoms  (136:15) | 4.96 ± 14.62 | 4.87 ± 11.90 | 0.000 | 0.990 |
| Change in GAF disability  (145:15) | 6.49 ± 16.21 | 2.33 ± 9.85 | 1.191 | 0.277 |

| NMDAR ANTIBODY (IgG; LIVE ASSAY) |  |  |  |  |
| --- | --- | --- | --- | --- |
| Change in GAF symptoms  (141:10) | 5.43 ± 13.97 | -1.70 ± 18.29 | 2.520 | 0.115 |
| Change in GAF disability  (150:10) | 6.77 ± 14.94 | -4.00 ± 23.75 | 4.234 | **0.041** |

Supplementary table 8: Change in GAF scores by NMDAR antibody serostatus (descriptive means shown with F and p values from ANOVA)

Abbreviations: NMDAR: N-methyl-d-aspartate receptor, GAF: global assessment of functioning.

| Diagnosis (data available on n subjects) | Antibody positive fixed CBA (n=11) | Antibody negative fixed CBA | p | Antibody positive live CBA (n=13) | Antibody negative live CBA | p |  |
| --- | --- | --- | --- | --- | --- | --- | --- |
| Current depressive episode (247) | 5 (45.5) | 77 (32.6) | 0.513 | 9 (69.2) | 73 (31.2) | **0.012** |  |
| Current manic episode (239) | 0 (0.0) | 1 (0.4) | 1.000 | 0 (0.0) | 1 (0.4) | 1.000 |  |
| Major depressive disorder (232) | 5 (50.0) | 105 (47.3) | 1.000 | 9 (75.0) | 101 (45.9) | 0.073 |  |
| Dysthymic disorder | 0 (0.0) | 16 (7.1) | 1.000 | 1 (9.1) | 15 (6.6) | 0.544 |  |
| Bipolar disorder I (235) | 1 (9.1) | 8 (3.6) | 0.355 | 0 (0.0) | 9 (4.0) | 1.000 |  |
| Bipolar disorder II (233) | 0 (0.0) | 9 (4.0) | 1.000 | 0 (0.0) | 9 (4.1) | 1.000 |  |
| Panic disorder (237) | 2 (18.2) | 57 (25.2) | 0.736 | 5 (41.7) | 54 (24.0) | 0.179 |  |
| Social phobia (238) | 1 (10.0) | 47 (20.6) | 0.692 | 2 (16.7) | 46 (20.4) | 1.000 |  |
| Specific phobia (239) | 2 (18.2) | 28 (12.3) | 0.633 | 1 (8.3) | 29 (12.8) | 1.000 |  |
| Generalized anxiety disorder (235) | 1 (9.1) | 25 (11.2) | 1.000 | 2 (16.7) | 25 (10.8) | 0.628 |  |
| Obsessive-compulsive disorder (221) | 2 (20.0) | 20 (9.5) | 0.261 | 2 (18.2) | 20 (9.5) | 0.301 |  |
| Body dysmorphic disorder |  |  |  |  |  |  |  |
| Post-traumatic stress disorder (237) | 0 (0.0) | 27 (11.9) | 0.619 | 2 (16.7) | 25 (11.1) | 0.633 |  |
| Somatisation disorder (234) | 0 (0.0) | 3 (1.3) | 1.000 | 0 (0.0) | 3 (1.3) | 1.000 |  |
| Adjustment disorder (234) | 0 (0.0) | 1 (0.4) | 1.000 | 0 (0.0) | 1 (0.5) | 1.000 |  |

Supplementary table 9: frequency of DSM-IV diagnoses according to NMDAR antibody serostatus. Diagnoses made using the Structured Clinical Interview for DSM-IV (SCID). Frequencies are given, numbers in brackets are the proportion of that serostatus group who met DSM-IV criteria for the specified diagnosis.

Abbreviations: CBA: cell-based assay, NMDAR: N-methyl-d-aspartate receptor.

|  | Antibody negative (fixed CBA) | Antibody positive (fixed CBA) | Antibody negative (live CBA) | Antibody positive (live CBA) |
| --- | --- | --- | --- | --- |
| AVLT IR total | Rho = .019  p = .815  n = 157 | Rho = -.661  p = .106  n = 7 | Rho =.000  p = .996  n = 155 | Rho = -.166  p = .669  n = 9 |
| WAIS estimated total IQ | Rho =.043  p = .526  n = 219 | Rho =.189  p = .626  n = 9 | Rho =.040  p = .559  n = 216 | Rho = -.004  p = .991  n = 12 |
| BPRS total | **Rho = -.130**  **p = .054**  **n = 219** | Rho = -.202  p = .576  n = 10 | **Rho = -.166**  **p = .014**  **n = 217** | **Rho =.590**  **p = .043**  **n = 12** |
| CAARMS total | **Rho = -.205**  **p = .004**  **n = 194** | Rho =.062  p = .885  n = 8 | **Rho = -.226**  **p = .002**  **n = 190** | Rho =.550  p = .064  n = 12 |
| BPRS positive sx | **Rho = -.150**  **p = .026**  **n = 220** | Rho = -.168  p = .643  n = 10 | **Rho = -.158**  **p = .020**  **n = 218** | Rho =.160  p = .620  n = 12 |
| SANS | Rho = -.103  p = .139  n = 206 | Rho =.012  p = .977  n = 8 | Rho = -.123  p = .081  n = 202 | **Rho =.620**  **p = .031**  **n = 12** |
| YMRS | **Rho = -.154**  **p = .022**  **n = 222** | Rho = -.055  p = .879  n = 10 | **Rho = -.144**  **p = .032**  **n = 221** | Rho = -.072  p = .834  n = 11 |
| MADRS | **Rho = -.177**  **p = .008**  **n = 226** | Rho = -.126  p = .728  n = 10 | **Rho = -.186**  **p = .005**  **n = 224** | Rho =.570  p = .053  n = 12 |

Supplementary table 10: Associations between cognitive/symptom scores and S100B levels by NMDAR antibody serostatus

Abbreviations: S100B: S100 calcium-binding protein B, NMDAR: N-methyl-d-aspartate receptor, AVLT: Rey Auditory Verbal Learning Task, IR: immediate recall, CAARMS: Comprehensive Assessment of At-Risk Mental States, WAIS: Wechsler Adult Intelligence Scale III, BPRS: Brief Psychiatric Rating Scale, SANS: Scale for the Assessment of Negative Symptoms, YMRS: Young Mania Rating Scale, MADRS: Montgomery-Asberg Depression Rating Scale

|  | Antibody positive | Antibody negative | F (serostatus) | p (serostatus) |
| --- | --- | --- | --- | --- |

| Neuronal surface antibody (any antigen; IgG, IgA, IgM; FIXED ASSAY) |  |  |  |  |
| --- | --- | --- | --- | --- |
| Amygdala bilateral  (11:148) | 3708.15 ± 412.53 | 3528.79 ± 407.31 | 3.961 | **0.048** |
| Hippocampus bilateral)  (11:148) | 8417.65 ± 828.97 | 8405.90 ± 780.99 | 0.083 | 0.773 |

Supplementary table 11: Limbic volumes (voxels) by neuronal antibody serostatus. Numbers in brackets under variable names are the number of seropositive subjects and seronegative subjects for whom data were available for that variable.
